# Fibrin Glue-Kartogenin Complex Promotes the Regeneration of the Tendon-Bone Interface in Rotator Cuff Injury

**DOI:** 10.1155/2021/6640424

**Published:** 2021-03-26

**Authors:** Jun Zhu, Jiahua Shao, Yi Chen, Guangyi Zhao, Lexiang Li, Qiwei Fu, Qirong Qian, Qi Zhou, Zheru Ding, Yiqin Zhou

**Affiliations:** ^1^Department of Orthopedics, Shanghai Changzheng Hospital, Naval Medical University, Shanghai 200003, China; ^2^Department of Pathology, School of Medicine, University of Pittsburgh, Pittsburgh, PA 15260, USA

## Abstract

**Objective:**

Rotator cuff injury healing is problematic because the tendon-bone junction often forms cicatricial tissues, rather than fibrocartilage, which leads to mechanical impairment and is prone to redamage. Kartogenin (KGN) is a newly discovered small molecule compound which can induce cartilage formation through chondrogenesis of endogenous mesenchymal stem cells.

**Methods:**

In this study, we used KGN with fibrin glue (FG) to repair the rotator cuff injury by promoting the formation of fibrocartilage at the tendon to bone interface. Firstly, we assessed the release rate of KGN from the FG-KGN complex and then created a rabbit rotator cuff tendon graft-bone tunnel model. The rabbits received saline, FG-KGN, or FG injections onto the tendon to bone interface after injury. Shoulder tissues were harvested at 6 and 12 weeks, and the sections were stained with HE and Safranin O/Fast green. The samples were assessed by histologic evaluation and biomechanical testing. Synovial mesenchymal stem cells derived from the synovial tissue around the rotator cuff were harvested for western blotting and qRT-PCR analysis.

**Results:**

KGN was released rapidly from the FG-KGN complex during first 4 hrs and followed by a slow release until 7 days. The tendon graft-bone interface in the control (saline) group and the FG group was filled with scar tissue, rather than cartilage-like tissue, and only a small number of chondrocytes were found at the adjacent bone surface. In the FG-KGN group, the tendon to bone interface was fully integrated and populated by chondrocytes with proteoglycan deposition, indicating the formation of fibrocartilage-like tissues. At 12 weeks, the maximum tensile strength of the FG-KGN group was significantly higher than that of the FG and control groups (*P* < 0.01). The RNA expression levels of tendinous genes such as Tenascin C and the chondrogenic gene Sox-9 were substantially elevated in SMSCs treated with the FG-KGN complex compared to the other two groups.

**Conclusion:**

These results indicated that fibrin glue is an effective carrier for KGN, allowing for the sustained release of KGN. The FG-KGN complex could effectively promote the regeneration and formation of fibrocartilage tissue of the tendon-bone interface in the rabbit rotator cuff tendon graft-bone tunnel model.

## 1. Introduction

Rotator cuff injury is the most common cause of shoulder dysfunction in clinical patients, causing long-term pain and limited movement [1]. Despite continuous progress in rotator cuff repair surgery, current treatment outcomes are still far from optimal. The recurrence rate of rotator cuff tear after surgical reconstruction ranges from 20%~94% [1–4]. The tendon-bone interface is a highly specific transition zone, which is made up of four layers: tendon, uncalcified fibrocartilage, calcified fibrocartilage, and bone [4, 5]. However, pathological study shows that the tissue structure of the rotator cuff after reconstruction cannot be restored to the original composition [6, 7]. The main problem is that the fibrocartilage in the tendon graft-bone interface fails to regenerate and is replaced by scar tissue [5]. As a result, its mechanical properties are impaired, which leads to deficient restoration of shoulder function and high recurrence rates of tendon-bone interface rupture. Therefore, fibrocartilage regeneration is the most significant challenge in rebuilding the tendon-bone interface and restoring shoulder function after injury since it is extremely difficult to regenerate a natural-like fibrocartilage. In recent years, researchers have used a variety of biological techniques to promote the regeneration of fibrocartilage, attempting to restore the tissue morphology and composition of the normal tendon graft-bone interface. There are currently four major treatment methods: mechanical stress stimulation [8], cell growth factor therapy [9, 10], mesenchymal stem cell therapy [11], and biomaterial enhancement [12]. Although most of the results from these methods reported cartilage regeneration and improved tensile strength, the mechanical properties were not fully restored, and some methods have not been approved for clinical application. Therefore, more effective and convenient biological intervention methods to promote rotator cuff tendon graft-bone interface healing are needed. Kartogenin (KGN) is a newly discovered synthetic small molecule compound [13] that can induce endogenous mesenchymal stem cells (MSCs) to differentiate into chondrocytes. It was initially identified in osteoarthritis cartilage repair studies [13]. The unique feature of KGN is that it does not require the addition of exogenous seed cells and scaffolds, which differs from the traditional tissue engineering cartilage regeneration practices. Studies have shown that KGN can also promote collagen fibril organization in the tendon-bone interface [14]. Therefore, KGN has a positive effect on cartilage and tendon regeneration, which provides an important foundation for the application of KGN in tendon graft-bone healing. In addition, MSCs from the adjacent synovium and tendon are involved in promoting tendon graft-bone healing following rotator cuff injury [15–17]. Therefore, these autologous MSCs are likely to differentiate into cartilage and synthesize cartilage matrix under KGN induction, promote cartilage regeneration at the tendon graft-bone healing interface, and ultimately improve the quality of tendon graft-bone healing. The purpose of this study is to explore whether KGN can promote the regeneration of cartilage at the interface of tendon graft-bone healing in a rabbit rotator cuff tendon graft-bone tunnel model as a novel intervention.

## 2. Methods and Materials

### 2.1. Ethics Statement

The protocol for use of rabbits in this study was approved by Navy Medical University Animal Care and Use Committee. Pain and discomfort are minimized based on standard animal care guidelines.

### 2.2. Preparation of FG-KGN Complex

KGN (Sigma-Aldrich, Catalog #SML0370) was dissolved in dimethyl sulphoxide (DMSO) to make a stock solution at a concentration of 100 mmol/l and diluted to 5 mmol/l with PBS as working solutions. Fibrin glue served as the carrier of KGN. To prepare the FG-KGN complex, 20 *μ*l of the 5 mM KGN solution was mixed well with the two components of fibrin glue (total volume 1 ml) and rested for 30 seconds at room temperature. The mixture formed a transparent hydrogel, termed the FG-KGN complex.

### 2.3. Determining KGN Release from FG-KGN Complex *In Vitro*

In order to measure the release rate of KGN from the FG-KGN complex, the following tests were performed. First, 0.5 ml of the FG-KGN complex containing 300 *μ*mol KGN was incubated in 15 ml tubes containing 10 ml PBS in an incubator at 37°C for 7 days. 0.2 ml PBS was collected at different time points (1 hour, 2 hours, 4 hours, 8 hours, 1 day, 2 days, 4 days, and 7 days) to determine the concentration of KGN in the PBS solution. After each 0.2 ml PBS was removed, an equal amount of fresh PBS was added to keep the volume constant. The amount of KGN in the PBS samples was measured by high performance liquid chromatography (HPLC) as described previously [18]. This experiment was repeated three times.

### 2.4. Creating Rabbit Rotator Cuff Injury Model

A rabbit rotator cuff injury model was created on the right foreleg of 72 4-6 month female New Zealand white rabbits. All rabbits were divided into three randomized groups: the saline group (control group), the fibrin glue-only group (FG group), and the FG-KGN complex implant group (FG-KGN group). The surgical procedure was performed as described below. All rabbits were anesthetized with 3% pentobarbital sodium (1 ml/kg) through an ear vein during the operation and placed in the lateral decubitus position. We then exposed the attachment of the supraspinatus tendon to the greater tuberosity of the humerus in the right shoulder and completely severed it. A 5 mm longitudinal, 1 mm wide, and 2 mm deep groove was then drilled in the lateral edge of the articular surface of the greater tuberosity. Two cross bone tunnels were made in the greater tuberosity by using a towel forceps through the bone groove, and the diameter of the two bone tunnels was 1 mm. The entrances of the two bone tunnels were at the both ends of the bone groove, and the exits were located on the outer surface of the great tuberosity. KGN (100 *μ*l, 100 *μ*mol/L) was mixed with 100 *μ*l fibrin glue and immediately injected into the bone groove, waiting for about 30 seconds for the fluid in the groove to cure. Then, the distal end of the supraspinatus tendon was sutured with nonabsorbable polypropylene suture (3-0) with a modified Mason-Allen. The tendon was pulled back to the bone groove carefully and fixated on the greater tuberosity. After cleaning the operation field, the skin was closed with nonabsorbable sutures. All rabbits were allowed free movement after surgery and had routine intramuscular injection of gentamicin (2.5 mg/kg) once a day for 3 days to prevent infection.

### 2.5. Histologic Evaluation

Three rabbits of each group were euthanized at 6 and 12 weeks, and the humerus was harvested at 2 cm below the humeral head with the distal end of the supraspinatus tendon. The specimens were immediately fixed in 10% formalin for 24 hours at room temperature and decalcified in 10% EDTA for 6 weeks. Needle testing of the specimens was conducted every 2 weeks until the needle could successfully pierce the tissue, indicating that they were completely decalcified. After decalcification, the specimens were dehydrated, embedded in paraffin, and cut to 6 *μ*m sections. The sections were stained with HE and Safranin O/Fast green, and were analyzed under a light microscope to evaluate tendon graft-bone healing. Additionally, the HE slides are subjected to polarized light microscopic observations of organized collagen fibre bundles and morphometric analyses. The sections were blindly scored using a modified tendon maturing scoring system [19].

### 2.6. Biomechanical Testing

To assess the strength of the repaired rotator cuff, we conducted a biomechanical test. The right forelegs from the three groups (six rabbits/group) were removed at 6 weeks and 12 weeks postoperatively for a biomechanical experiment. The humerus was cut off at 5 cm below the humeral head, and the scapula was amputated at 5 cm away from the glenohumeral joint. Surrounding soft tissues were completely removed to avoid associated confounds. Biomechanical testing (maximum tensile strength) was performed on a material testing machine (Instron 3345) with a standard testing program (Bluehill 2.0).

### 2.7. Isolation and Culture of Synovial Mesenchymal Stem Cells

Three rabbits of each group were euthanized at 6 and 12 weeks. Rabbit shoulder synovial mesenchymal stem cells (SMSCs) were isolated and cultured following a previously described protocol [20]. Briefly, the synovial tissue around the rotator cuff was harvested after surgery, and adipose and fibrous connective tissues were removed in the culture dish. The synovial tissue was then cut into 1-2 mm^3^ fragments with tissue scissors, and 4 ml 0.4% collagenase I (Gibco, Catalog #17100017) was added to initiate a 4 hr digestion in the 37°C incubator. After filtration with a 120-mesh nylon net (70 *μ*m), the filtrate was centrifuged at 400 g for 10 min, and the supernatant was discarded. The procedure was repeated three times, and the cells were suspended in PBS and counted. The isolated synovial cells were cultured at 37°C and 5% CO_2_ condition in the basic culture medium (special culture medium for mesenchymal stem cells, 5% nutritional additive GROTM, 100 U/ml penicillin, and 100 U/ml streptomycin). The growth of the cells was monitored every day, and primary cells were passaged after 7-10 days, then cultured and passaged at 80% confluency. Passage 3 (P3) SMSCs were used for subsequent experiments. Detection of synovial mesenchymal stem cell surface markers followed a previously described protocol [20].

### 2.8. Western Blotting and qRT-PCR Analysis

Synovial mesenchymal stem cells (P3) were treated with RIPA lysis buffer for extracting proteins. A BCA assay was used to determine the protein concentration. The samples, each containing 50 *μ*g protein lysate, were loaded for sodium dodecyl sulfate-polyacrylamide gel electrophoresis (SDS-PAGE) and transferred onto nitrocellulose membranes after 120 V 1 h electrophoresis. After 1 hr blocking with 5% BSA, the membranes were incubated with primary antibodies including anti-Tenascin C, anti-Sox-9, anti-PPAR-*γ*, anti-Runx-2, and anti-OCT-4 at a dilution of 1 : 1000, and GAPDH 1 : 1000. The immunoreactive bands were scanned and analyzed with ImageJ software (National Institutes of Health, Bethesda, MD, USA).

Total RNA of synovial mesenchymal stem cells was extracted by using the TRIzol Reagent, according to the manufacturer's instructions. RNA was reverse transcribed with the SuperScript™ Preamplification System. The following genes were used as internal controls in PCR amplification and their expression was assessed: Tenascin C, Sox-9, PPAR-*γ*, Runx-2, OCT-4, GAPDH. The primer pair sequences were as follows: Oct-4, 5′-CGA GTG AGA GGC AAC TTG G-3′, 5′-CGG TTA CAG AAC CAC ACA CG-3′; Sox-9, 5′-TGA ATC TCC TGG ACC CCT TC-3′, 5′-CCG TTC TTC ACC GAC TTC CT-3′; Runx-2, 5′-TGA TGA CAC TGC CAC CTG TG-3′, 5′-ACT CTG GCT TTG GGA AGA GC-3′; PPAR-*γ*, 5′-TGC AGG AGC AGA GCA AAG AAG-3′, 5′-GAG GCC AGC ATG GTG TAG ATG-3′; Tenascin C, 5′-CGTGAAAAACAATACCCGAGGC-3′, 5′-GCCGTAGGAGAGTTCAATGCC-3′; and GAPDH, 5′-AAG GCC ATC ACC ATC TTC CA-3′, 5′-GGA TGC GTT GCT GAC AAT CT-3′. All samples were assayed in triplicate.

## 3. Results

### 3.1. KGN Release from the FG-KGN Complex *In Vitro* In a Gradual Manner

**Figure 1:**
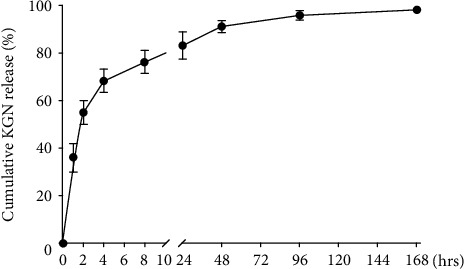
KGN release rate from the FG-KGN complex. Within 2 hours, the release rate of KGN was high, reaching about 60% of the total release amount. The release rate gradually decreased at 4 hours, and then remained steady between 4 and 96 hours. At 96 hours, KGN was mostly released from the FG-KGN complex.

As the FG-KGN complex soaked in PBS, within 2 hours, the release rate of KGN was high, reaching about 60% of the total release amount. The release rate gradually decreased at 4 hours, and then remained steady between 4 and 96 hours. At 96 hours, KGN was mostly released from the FG-KGN complex. These results indicate that KGN is rapidly released from the FG-KGN complex within the first four hours, then slowly released over the next few days (Figure 1).

### 3.2. Histologic Evaluation Results

**Figure 2:**
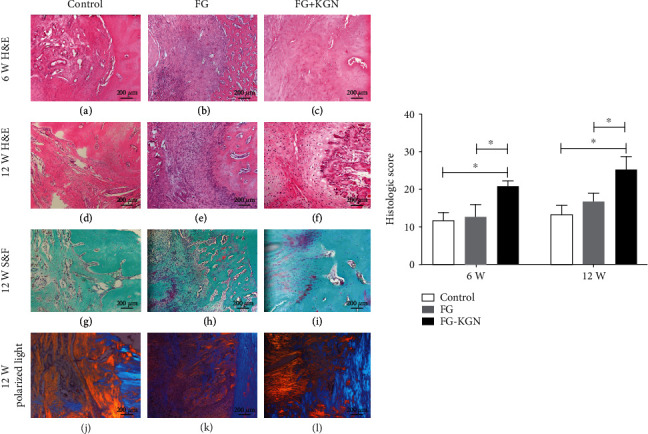
The tendon graft-bone interface healing at 6 and 12 weeks posttreatment. The tissue specimens were analyzed by HE staining (a–f), Safranin O/Fast green staining (g–i), and polarized light (j–l). At 6 and 12 weeks, the histological scores in the FG-KGN group were significantly higher than those in the control group and the FG group (*P* < 0.01). Asterisks show a significant difference between groups (*P* < 0.01). Bar: 200 *μ*m.

At 6 weeks postsurgery, a disordered structure was observed in the control groups (Figure 2(a)), and there were obvious gaps between the bone and the tendon tissue. No obvious formation of fibrocartilage was observed. The FG group had a relatively orderly structure (Figure 2(b)); no obvious gap was observed between the bone and tendon tissues, but little fibrocartilage was found in the tendon graft-bone interface. In the FG-KGN groups, the tissue structure was orderly (Figure 2(c)); there was no obvious gap between the bone and tendon, and a small amount of fibrocartilage tissue was formed. At 12 weeks after the operation, we observed relatively orderly structures in the control groups (Figure 2(d)); there were few gaps between the bone and tendon tissue, but no obvious cartilage formation was observed. The FG group had an orderly structure and no gap (Figure 2(e)); a small amount of cartilage was formed, and chondrocytes were visible. In the FG-KGN group (Figure 2(f)), the regenerated tissue structure was orderly, with no gaps. Cartilage was formed at the interface, and the arrangement was regular. The results of Safranin O/Fast green staining confirmed that the FG-KGN group showed obvious Safranin O-positive staining (Figure 2(i)), suggesting that cartilage was regenerated and arranged in a fibrous shape, similar to the structure of fibrocartilage. In the control and FG groups, there was no or only minimal fibrocartilage formation, and an obvious boundary between bone and tendon can be seen (Figures 2(g) and 2(h)). Additionally, in the polarized light experiment, it was observed that collagen fibers were disorderly arranged and did not have a regular structure in the control group and FG group (Figures 2(j) and 2(k)). In the FG-KGN group, the collagen fibers were orderly arranged, which indicated the proliferation of fibrocartilage (Figure 2(l)). At 6 and 12 weeks, the histological scores in the FG-KGN group were significantly higher than those in the control group and the FG group. (Figure 2, *P* < 0.01).

### 3.3. Biomechanical Testing Results

**Figure 3:**
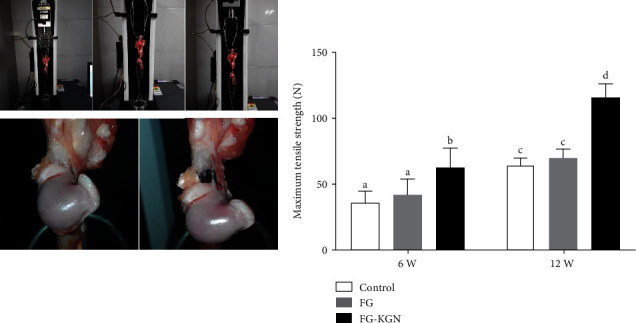
Biomechanical properties of the repaired rotator cuff were tested by a mechanical tensile test. At 12 weeks, the maximum tensile strength of the FG-KGN group was significantly higher than that of the control group and the FG group (P<0.01). Treatment with FG alone, however, did not significantly increase the strength over the control group.

At 12 weeks, the maximum tensile strength of the FG-KGN group was significantly higher than that of the control group and the FG group (*P* < 0.01), and the maximum tensile strength (115.70 ± 10.15 MPa) of the FG-KGN group was almost twofold that of the control group (64.52 ± 5.29 MPa) and the FG group (69.74 ± 6.58 MPa). Treatment with FG alone, however, did not significantly increase the strength over the control group (Figure 3). Although the mechanical properties of the tendon-bone interface in the FG-KGN group were significantly higher than that in the FG group and the control group, the results still did not reach the level of healthy tissues.

### 3.4. qRT-PCR and Western Blot Results

**Figure 4:**
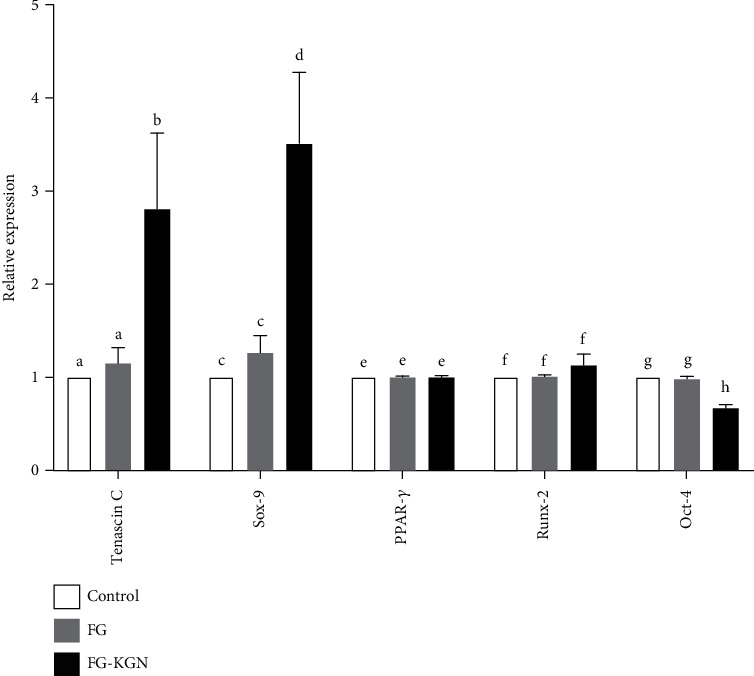
The qRT-PCR results. The expression levels of Tenascin C and Sox-9 was were highly elevated in SMSCs treated with the FG-KGN complex, and were more than twice compared to those in the other two groups (P<0.01). The expression levels of Oct-4 in the FG-KGN group wereas significantly lower than that those in the other two groups, which indicated that SMSCs were differentiated after treatment with FG-KGN. (P<0.01). No significant differences were observed in tThe expression levels of PPAR-*γ* and Runx-2 were observed non significant difference between the FG-KGN treatment group and the other onesgroups (P>0.05).

The qRT-PCR results indicate that the expression level of the tendinous gene Tenascin C was highly elevated in SMSCs treated with the FG-KGN complex and was more than twofold compared to the other two groups (*P* < 0.01). The chondrogenic gene Sox-9 was also significantly upregulated in SMSCs treated with the FG-KGN complex compared with the control and FG groups (*P* < 0.01). The expression levels of the lipogenic gene PPAR-*γ* and the osteogenic gene Runx-2 were assessed, and there were no significant differences between the FG-KGN treatment and the controls (*P* > 0.05). The expression of the stemness marker OCT-4 in the FG-KGN group was slightly lower than the other two groups, but this was not statistically significant (*P* > 0.05, Figure 4).

**Figure 5:**
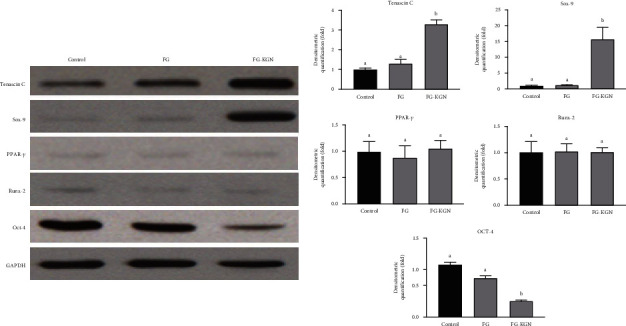
The western bolt results. There was non significant difference in the expression of each protein between the FG group and the control group, while the expression of Tenascin C and Sox-9 in the FG-KGN group was significantly higher than that in the other two groups (P<0.01). The expression level of OCT-4 in the FG-KGN group was lower than that in the other two groups (P<0.01).

Western blot results revealed no significant difference in the expression of each protein between the FG group and the control group, while the expression of Tenascin C and Sox-9 in the FG-KGN group was significantly higher than the other two groups (*P* < 0.01, Figure 5). The expression level of OCT-4 in the FG-KGN group was lower than that in the other two groups (*P* < 0.01, Figure 5). These results indicate that with FG-KGN treatment, the expression levels of genes and proteins related to tenogenesis and chondrogenesis (Tenascin C and Sox-9) were significantly increased while the stemness of SMSCs obviously decreased. There were no significant changes in lipogenic and osteogenic proteins and genes (PPAR-*γ* and Runx-2), indicating that FG-KGN can promote the regeneration of the fibrocartilage in tendon graft-bone interface without inducing additional osteogenesis or adipogenesis.

## 4. Discussion

In this study, we used fibrin glue as a drug carrier which transported KGN in the form of a FG-KGN complex and implanted KGN at the tendon graft-bone healing interface. Prior to surgical intervention, we assessed the release rate of KGN from the FG-KGN complex *in vitro*, which demonstrated that KGN is gradually released from fibrin glue to act on the surrounding microenvironment. After creating a rotator cuff tendon graft-bone tunnel model and applying FG-KGN *in vivo*, clusters of chondrocytes appeared at the tendon graft-bone healing interface in the FG-KGN group at 6 weeks after the operation, while fibroblasts were still predominant in the control group. At 12 weeks, the tendon graft-bone interface tissue in the FG-KGN group was best integrated, and the chondrocytes were aligned in groups and more differentiated. Some of the chondrocytes near the bone surface had mineralized and formed a characteristic tidal line structure, indicating that the tendon graft-bone interface had healed better than the control group, which was mainly composed of scar tissue with a few chondrocytes. Biomechanical testing indicated that the maximum tensile strength of the tendon graft-bone interface healing in the FG-KGN group was significantly higher than that in the FG and control groups at both the 6- and 12-week time points. At 12 weeks postsurgery, the maximum tensile strength of the FG-KGN group was nearly twice that of the control or FG group. The results indicate that KGN not only promoted cartilage-like tissue regeneration at the tendon graft-bone interface, it also increased the interface strength significantly. KGN can also promote the formation of new tissue at the interface, which increased the thickness of the interface tissue and made it more resistant to rupture. Similarly, the maximum tensile strength of the new tissue of the tendon graft-bone interface healing in the KGN group was significantly higher than that in the control group, indicating that the repaired tissue in the KGN group had increased resilience and greater resistance to deformation, further indicating that KGN improved the quality of tendon graft-bone healing.

The synovial mesenchymal stem cell was one of the main MSCs in the shoulder or other joints, and it was reported that the synovial mesenchymal stem cell could join in the early remodeling of tendon-bone healing [21, 22]. In our study, synovial mesenchymal stem cells were isolated from a rabbit shoulder after a different treatment; they were used for RNA expression and protein production analysis to prove the effect of KGN intervention. Our results showed that the tendon- and cartilage-related markers of synovial mesenchymal stem cells were significantly increased while stemness declined after KGN treatment, which means that KGN treatment could alter the phenotype of synovial mesenchymal stem cells of a rabbit shoulder and induce them to differentiate into fibrochondrocytes.

Kartogenin (KGN) is a small molecule compound selected from more than 22,000 heterocyclic drug molecules by Johnson et al. [13]. It can effectively promote the differentiation of mesenchymal stem cells into chondrocytes. So, KGN was widely used for articular cartilage repair studies recently [18, 23–25]. However, its application in tendon-bone interface healing was infrequent. In another preclinical study, Zhang and Wang [26] injected KGN directly into an Achilles tendon injury in rats and observed fibrocartilage formation at the injection site after 2 weeks by histology evaluation. Wang et al. [14] recently used KGN for rotator cuff injury healing and also concluded that KGN could promote cartilage regeneration in a murine model. However, they had only observed for 4 weeks and did not differentiate whether the fibrocartilage or hyaline cartilage was regenerated at the tendon-bone interface. Our previous study also proved that KGN could induce fibrocartilage regeneration in the rat tendon-bone tunnel interface healing model which had improved both Col-1 expression and Col-2 expression [27]. In this study, we proved the KGN also works in a rabbit rotator cuff tendon-bone graft tunnel model by improving both Tenacin C and Sox-9 expression and inducing fibrocartilage regeneration. It is worth mentioning that Yuan et al. [28] used KGN to create an animal model of tendinopathy because of its strong ability to induce MSC chondrogenic differentiation. This means that direct injection of the KGN solution into a joint is not safe. It is better to use a carrier to localize KGN in the injury site.

Our research still has some limitations. For the *in vivo* experiments, we only used a single dose of KGN and did not study the effect of different concentrations of KGN on the regeneration of the tendon graft-bone interface. In addition, we did not compare the effects of KGN with other methods (e.g., mesenchymal stem cells and cytokines) on the healing of the rotator cuff injury. At the same time, since KGN promotes the healing of the tendon graft-bone interface by inducing differentiation of mesenchymal stem cells into chondrocytes, mesenchymal stem cells could be combined with KGN and may lead to even better healing outcomes. It is worth mentioning that the tendon graft-bone tunnel model is not a perfect representation of the clinical scenario of rotator cuff injury. In a future study, we will focus on creating a better rotator cuff injury model. At the same time, the release rate of KGN could be further optimized with specific biomaterial to render a more sustained release pattern.

The FG-KGN complex has a certain viscosity and can be attached to a specific location, which is conducive to the repair of injured parts. Also, fibrin glue did not release a large number of growth factors such as PRP; thus, we could clearly figure out the effect of KGN only. In the follow-up experiments, we plan to compare these carriers to choose the optimal treatment.

## 5. Conclusion

In the current study, we observed that KGN could promote fibrocartilage regeneration at the interface of rotator cuff tendon graft-bone healing, restore the injured rotator cuff with better morphology, and improve the mechanical strength of the tendon graft-bone healing interface, indicating comprehensive improvement in the quality of tendon graft-bone healing. It is suggested that this intervention could reduce the recurrence rate of rotator cuff tears and has substantial clinical application value. We also demonstrated that fibrin glue is an effective drug carrier for KGN, allowing for the sustained release of KGN. Further studies to clarify the possible molecular mechanism of KGN-induced cartilage regeneration at the tendon graft-bone interface are required for better application of this intervention.

## Data Availability

All data is available from the corresponding author Yiqin Zhou (drzhouyiqin@163.com) upon request.
